# A conspiracy of glioma and endothelial cells to invade the normal brain

**DOI:** 10.18632/oncotarget.219

**Published:** 2011-02-18

**Authors:** Abdessamad Zerrouqi, Erwin G Van Meir

**Affiliations:** ^1^ Laboratory of Molecular Neuro-Oncology, Department of Neurosurgery, Emory School of Medicine, Emory University, Atlanta, GA, USA; ^2^ Departments of Hematology and Medical Oncology, Emory School of Medicine, Emory University, Atlanta, Georgia, USA; ^3^ Winship Cancer Institute, Emory University, Atlanta, Georgia, USA

Gliomas are the most common primary tumors of the central nervous system, accounting for the majority of adult primary brain tumors [1]. One of the major pathophysiological features of malignant human gliomas is their ability to diffusely and deeply invade into surrounding brain tissues [2-4]. The invasive phenotype is acquired early during glioma progression; even in low grade tumors, single or small groups of cells detach from the original site, attach to and remodel the extracellular matrix (ECM) during their migration, and penetrate and destroy adjacent brain structures [5]. The diffusely infiltrative nature of gliomas is believed to underlie tumor recurrence after surgery; therefore, understanding the mechanisms of glioma invasiveness and their response to therapy are very important.

To investigate these processes, initial work has focused on tumor-ECM interactions. The invasive process of glioma cells requires changes in the adhesive properties of the cells and expression of proteases. Neural cell adhesion molecules [6] and E-cadherin [7] are downregulated, while CD44 [8] and N-cadherin [7, 9, 10] are upregulated. Attachment to ECM involves integrins such as β1-integrin [11-13] and tenascin-C [14]. ECM remodeling is accomplished via the action of several classes of proteases including matrix metalloproteinases (MMPs), [2], ADAMs (a disintegrin and metalloproteinase), plasmin and cathepsins (2). Invasion also requires cytoskeletal rearrangements, formation of focal adhesions and lamellipodia involving Rho and Rac [15].

The human Angiopoietin/Tie system plays a crucial role in the remodeling of the tumor microenvironment that accompanies the angiogenic switch in tumors, in order to control endothelial cell survival and vascular maturation and thereby promoting tumor growth.

The Angiopoietin family includes four ligands (Angiopoietins-1, 2, 3 and 4). The best characterized are Ang1 and 2, while the role of Ang3 and Ang4 are less well known. These ligands bind to the Tie1 and Tie2 tyrosine kinase receptors. Tie2 is well studied and expressed by endothelial cells, hematopoietic cells and tumor cells, and binds both Ang-1 and Ang-2 with similar affinity. In contrast, Tie1 is an orphan receptor, exclusively expressed by endothelial cells (ECs) whose role is incompletely defined [16].

Ang-1, secreted by perivascular cells and pericytes, acts in a paracrine agonistic manner and induces endothelial Tie2 phosphorylation and subsequent vessel stabilization. Ang1 binding to Tie2 results in receptor dimerization, which stimulates autophosphorylation of the Tie2 kinase domain [17], and triggers survival signaling in resting ECs by activating PI3kinase/AKT [18]. The signaling through PI3kinase promotes Ang1-induced cell survival, control of vascular permeability and regulates the capillary sprouting that occurs during normal and tumoral angiogenesis [19].

In contrast, Ang-2 acts as an antagonist of Ang-1-mediated Tie2 activation. Autocrine production of Ang-2 by endothelial cells serves as a rheostat to control the level of Tie2 phosphorylation by Ang1. Ang2 modulates the vascular remodeling occurring during angiogenesis in a cooperative manner with pro-angiogenic factors including vascular endothelial growth factor-A (VEGF-A) [20]. Ang2 dose-dependently inhibits Ang1-induced Tie2 phosphorylation, Akt activation, EC survival [21], and induces vessel destabilization and pericyte dropout at higher concentration. Ang-2 is strongly expressed in the vasculature of many tumors where it is believed to act synergistically with pro-angiogenic cytokines and promote tumor-associated angiogenesis and tumor progression. The elevation of expression of VEGF and Ang2 is believed to play a key role in the pseudopalisading necrosis and associated glomeruloid microvascular proliferation that are pathognomonic features of glioblastomas [22-24].

Although Tie2 was originally described as a specific receptor for endothelium, its expression has been reported in nonvascular normal and neoplastic tissues [12, 25, 26]. Tie2 was found expressed by hematopoietic stem cells [27], and in gastric [28], thyroid [29] and breast cancers [30]. The expression of Tie2 is particularly high in glioma as compared to normal brain tissue, and increases with the progression from low to high grade gliomas [12]. Tie2 activation can modify the adhesion properties of glioma cells by increasing their binding to ECM components such as collagen type I and IV through the induction of the expression of β1-integrin [12], similar to observations in hematopoietic stem cells [27].

**Fig 1:**
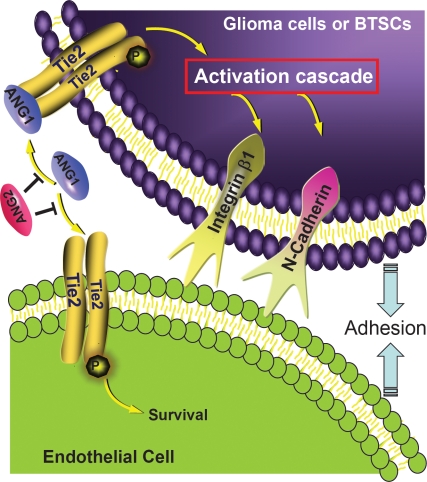
Tie2 activation enhances the interaction of glioma and brain tumor stem-like cells with endothelial cells and promotes their invasive phenotype Angiopoietin1 (Ang1) present in the extracellular matrix activates Tie2 that is expressed by glioma and brain tumor stem-like cells (BTSC) and thereby upregulates β1–integrin and N-cadherin. This upregulation increases cancer- endothelial cell interactions, which promote tumor cells to invade healthy brain tissue. Ang2 acts as antagonist of Ang1 and prevents Tie2 receptor activation.

A new study by Liu et al [31] in the recent December issue of *Oncotarget* broadens our knowledge of glioma cell invasion to interactions with other cells in the tumor environment. They propose that Tie2/Ang1 promotes the invasion phenotype by modulating the interaction of glioma and brain tumor stem like cells (BTSC) with endothelial cells. This cooperation was evidenced when Tie2-positive tumor cells were co-injected with endothelial cells in brains of immunocompromised mice. The tumor cells migrated farther from the injection site, developed multifocal tumors surrounding vascular structures and tended to be more aggressive than Tie2 negative-tumors. They determined that Ang1-mediated Tie2 activation in glioma cells and BTSCs enhances their adhesion to human umbilical vein endothelial cells *in vitro*. The mechanism underlying the latter effects involves the upregulation of β1-integrin and N-cadherin (Fig.1). While VEGF may regulate endothelial cell-cell or cell-matrix interactions [32], they found Ang1/Tie2 mediated adhesion to be VEGF independent. Tie2 also increased the tumor cells' autonomous invasion capabilities in a 3D matrix, but unfortunately, the influence of endothelial cells was not proposed that heterotypic cell contact/communication between cancer cells and adjacent vascular cells can boost the tumor invasive phenotype and thereby malignancy. These findings provide new insights on how expression of Tie2 on tumor cells is translated into a highly infiltrative phenotype in human gliomas.

The *in vivo* tumor phenomenon observed by Liu et al [31] is intriguing and raises further questions. How do tumor cells more efficiently invade normal brain tissue and establish tumor foci when they coexist with endothelial cells? Does this phenomenon require tumor cells to bind to and co-migrate with endothelial cells? Are endothelial cells providing paracrine pro-migratory or pro-survival signals for cancer stem-like cells? Do the endothelial cells participate in accelerating the formation of a vascular supply to the tumor? Clearly, the effect is Tie2-dependent, as it was not observed in parental tumor cells mixed with endothelial cells. A demonstration of this effect with brain endothelial cells and a careful analysis of their presence in clusters of migrating Tie-2- positive glioma cells and alteration of N-cadherin and β1-integrin should help answer this question and provide a link with the *in vitro* findings on adhesion.

What do these novel findings mean for therapy? Since this increase in malignancy of glioma cells is associated with Tie2 expression, the obvious strategy is to target the signaling emanating from Tie2 upon angiopoietin activation. A handful of drugs targeting Tie2 tyrosine kinase are under clinical evaluation (CEP-11981, CE-245677 and AMG-386). Another clinically relevant question is whether changes in the invasive phenotype seen in tumors of patients treated with anti-VEGF antibodies (Bevacizumab), are related to Tie2 upregulation and this VEGF-independent invasive mechanism. The results of the clinical trials conducted with these agents and a deeper mechanistic understanding of glioma–endothelial cell communication in invasion will significantly extend this knowledge to more effective clinical translation for the treatment of glioblastoma patients. We eagerly anticipate more to unfold in this interesting (com)plot.
